# Guidance for conducting and evaluating serological surveys to assess interruption of yaws transmission in the context of an eradication target

**DOI:** 10.1371/journal.pntd.0012899

**Published:** 2025-04-23

**Authors:** Oriol Mitjà, Katherine Gass, Michael Marks, Philip J. Cooper, Petter J. Diggle, Lance Waller, Patrick Agana-Nsiire, Belen Lardizabal Dofitas, Louise Dyson, Julie Jacobson, John Kaldor, Sung Hye Kim, Susana Vaz Nery, Chandrakant Revankar, Ghislain Sopoh, Anthony W. Solomon, Daniel Argaw Dagne, Priya Pathak, Aya Yajima, Zaw Lin, Mahoutondji Yves Thierry Barogui, Ronaldo Carvalho Scholte, Kazim Hizbullah Sanikullah, Chris Drakeley, Gillian Stresman, John Gyapong, Kingsley Bampoe Asiedu

**Affiliations:** 1 Fight Infectious Diseases Foundation, Hospital Germans Trias i Pujol, Badalona, Spain; 2 NTD Support Center, Task Force for Global Health, Decatur, Georgia, United States of America; 3 London School of Hygiene & Tropical Medicine, London, United Kingdom; 4 Escuela de Medicina, Universidad Internacional del Ecuador, Guayaquil, Ecuador; 5 Institute of Infection and Immunity, St George’s University of London, London, United Kingdom; 6 Lancaster Medical School, Lancaster University, Lancaster, United Kingdom; 7 Liverpool School of Tropical Medicine, Liverpool, United Kingdom; 8 Rollins School of Public Health, Emory University, Atlanta, Georgia, United States of America; 9 Anesvad Foundation, Accra, Ghana; 10 University of the Philippines, Manila, Philippines; 11 Mathematics Institute and School of Life Sciences, University of Warwick, Coventry, United Kingdom; 12 Bridges to Development, Vashon, Washington, United States of America; 13 Kirby Institute, UNSW, Sydney, New South Wales, Australia; 14 Hanyang University College of Medicine, Seoul, Republic of Korea; 15 Public health medical consultant, North Brunswick, New Jersey, United States of America; 16 Regional Institute of Public Health, University of Abomey—Calavi, Cotonou, Benin; 17 Global Neglected Tropical Diseases Programme, World Health Organization, Geneva, Switzerland; 18 World Health Organization Regional Office for South-East Asia, New Delhi, India; 19 World Health Organization Regional Office for Africa, Brazzaville, Congo; 20 Pan American Health Organization, Washington, District of Columbia, United States of America; 21 World Health Organization Regional Office for the Western Pacific, Manila, Philippines; 22 College of Public Health, University of South Florida, Tampa, Florida, United States of America; 23 Centre for Neglected Tropical Diseases Research, University of Health and Allied Sciences, Ho, Ghana; Institute of Continuing Medical Education of Ioannina, GREECE

## Abstract

This document provides a summary of guidance developed for national programmes on conducting serosurveys to assess yaws transmission status, with the objective of confirming yaws seroprevalence below 1% at each of three serosurveys over a period of 3–10 years after reporting the last case of active yaws in a region. It proposes active testing of children aged 1–5 years through population-based surveys and includes recommendations on survey design, sample size determination, sampling of primary sampling units (PSUs) within an evaluation unit, sampling of households within PSUs, integration with existing public health surveys, and follow-up protocols for positive results. Geospatial analysis and sustained surveillance are recommended for accurate assessment of whether transmission interruption has been achieved.

## Introduction

Yaws, a neglected tropical disease (NTD) caused by *Treponema pallidum* subsp. *pertenue*, predominantly affects children in low- to upper-middle-income countries in the tropics [1–3]. If untreated, yaws can lead to severe health problems, progressing from initial skin ulcers to secondary and tertiary stages, resulting in chronic disfigurement and disability.

The epidemiological status of yaws indicates its persistence as a significant public health issue in certain populations. According to World Health Organization (WHO) data, yaws is endemic in at least 16 countries, with most cases reported from WHO’s Western Pacific Region [4]. In 2023, 222,652 suspected yaws cases were reported to WHO from 13 countries, although only 1,477 cases were confirmed in 9 countries. About 85% of all suspected cases were reported from two countries: Papua New Guinea (100,528) and Indonesia (88,646). However, laboratory tests are not yet routinely performed to confirm cases, highlighting challenges in accurate diagnosis and reporting [4].

In the 2021–2030 NTD road map [5], endorsed by the World Health Assembly in November 2020 [6], yaws is targeted for global eradication by 2030. WHO’s comprehensive yaws eradication strategy includes the use of azithromycin mass drug administration (MDA) [7], which has proven its efficacy, effectiveness, and safety for reducing the prevalence of yaws at population level [8,9]. This approach requires more than 90% coverage of the whole population in each of three MDA rounds in high endemic areas [10–12]. Recommended post-MDA impact assessment includes both passive and active surveillance [13]. Routine passive surveillance, in which patients seek care for their illnesses at health facilities, detects active yaws (i.e., symptomatic cases), though most affected children do not seek treatment. Active surveillance, involving outreach activities, facilitates the identification of active yaws cases in communities and schools, as well as the implementation of serosurveys to identify latent yaws (i.e., asymptomatic seroreactors). The criteria established by WHO for verification of interruption of transmission include a 3-year period, beginning two years after the last reported case of yaws, with (i) no new serologically confirmed active yaws cases, (ii) no new PCR-confirmed cases, and (iii) no evidence of new transmission events based on serosurveys (i.e., latent or active yaws in children aged 1–5 years born after the last reported case) [14]. WHO’s strategy also emphasizes the integration of yaws eradication efforts within broader NTD or skin-disease-focused strategies, to enhance resource optimization, intervention coverage, and surveillance efficiency [13].

The yaws eradication strategy, as previously published [7,13,15], does not specify parameters for the required serosurveys. Inadequate investigation could lead to biases, resource misuse, or missed cases, the last increasing the risk for a rebound in community transmission of yaws. Recommendations summarized in this policy platform provide a framework for national programmes to conduct effective serosurveys to assess the local interruption of yaws transmission.

## Overview of the survey policy and objectives

In March 2023, the World Health Organization Global Neglected Tropical Diseases Programme (WHO/NTD) established an Informal WHO Working Group on Yaws Survey Protocol Development including independent experts from multiple disciplines. The group was asked to provide guidance for serological surveys to assess the interruption of transmission of yaws at national or local levels. This document summarizes the conclusions of the group.

Guidance was developed based on the WHO strategy for yaws eradication [7], statistical principles, expert opinion, and best practice, using the data and experiences available in 2023. Low-level transmission or absence of transmission should be demonstrated using seroprevalence surveys. These surveys must meet methodological standards to provide confidence for public health officials and policy-makers. Given that the agreed global public health target for yaws is eradication, a large sample size is encouraged wherever possible. It is not possible for a single survey to provide sufficient evidence to demonstrate the interruption of transmission at a local level or within any geographical region. Therefore, national programmes should aim to document multiple lines of evidence, including sequential surveys and evidence of active and passive surveillance being conducted. A single survey will most likely not be sufficiently powered to exclude low-level transmission, but sequential surveys over several years should provide the necessary level of confidence that local interruption of transmission has been achieved.

**Fig 1:**
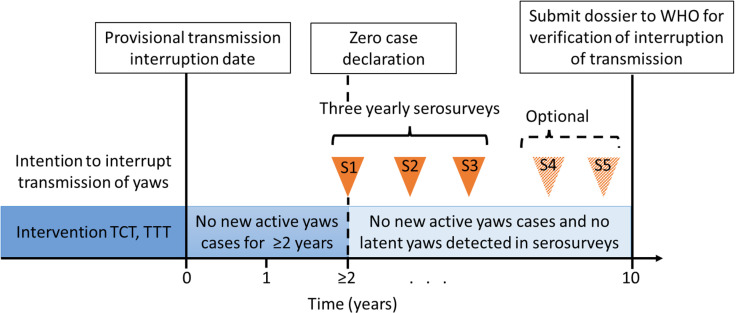
Overall roadmap of yaws eradication and scope of this policy guidance platform. TCT, total community treatment; TTT, total targeted treatment, S1, initial serosurvey; S2,= first follow-up survey; S3, second follow-up survey; S4, third follow-up survey (optional); S5, fourth follow up survey (optional).

Survey protocols recommended in this document rely on active enrollment and testing of 1–5-years-olds in population-based surveys using fingerpick blood sample (“serosurveys”). These surveys aim to measure whether the prevalence of latent-yaws cases in a specific area, called the EU, is below 1%. Serosurveys should start at least 2 years after the last reported active case of yaws (i.e., 2 years after the provisional transmission interruption date) as a result of successive rounds of MDA (Fig 1) and/or other interventions. At least three serosurveys conducted between 2 and 10 years after zero case declaration are necessary to provide confidence that a state of no observed yaws transmission has been maintained. In cases of large EUs or when initial surveys show prevalence above 1%, more than three surveys might be required.

In line with the strategic shift from vertical to integrated NTD programmes [6], yaws programmes are encouraged to conduct surveillance alongside other planned activities (such as surveys for other NTDs). As such, the guidance herein on survey design should be interpreted as broad guidance. Programmes are encouraged to find opportunities to incorporate yaws testing into existing surveys, in which case the sampling strategy and sample size can be adjusted to facilitate integration and maximize efficiency.

A Technical Advisory Group is recommended to be formed at the start of serosurveillance to guide the national program on the timing, design, and interpretation of these surveys. This group should include experts in yaws, epidemiology, clinical practice, and statistics. A definition of the Technical Advisory Group and other clinical and epidemiological terms employed in this policy guidance are provided in the S1 Appendix.

## Choice of serological tests

Table 1 lists various serological tests available for yaws, each of which has advantages and disadvantages [16–18]. None of these tests can distinguish yaws from syphilis; therefore, result interpretation may in some cases require careful clinical assessment [19]. The selection of diagnostic tools depends on availability, budget, and how well they fit with other disease control programs which act as platforms. Typically, all these tests have high accuracy when used on individuals suspected of having active yaws. However, in low-prevalence populations, particularly for the detection of latent cases in a post-MDA setting, they are prone to producing more false-positive results due to the lower positive predictive power. Box 1 summarizes the list of recommendations regarding the use of serological tests, including essential features for result interpretation.

**Box 1. Summary of key recommendations**

**1. Overall approach**

- Follow this guidance, which is based on expert opinion and accepted best practice for conducting high-quality seroprevalence surveys to demonstrate low or no transmission.
- Use yaws serosurveys with the aim of measuring latent yaws prevalence below 1% at evaluation units (EUs)-level, requiring at least three surveys conducted over 2–10 years to establish that there is high likelihood that transmission has not continued after the provisional transmission interruption date. Increase survey precision over time through follow-up surveys.
- Integrate yaws serosurveys with other activities like other disease control interventions, EPI/nutrition programs or surveillance work, whenever possible.

**2. Recommendations for yaws test selection**

- Ensure tests are performed with proper equipment by regularly trained personnel and proficiency testing is conducted for accuracy and competency.
- Employ rapid diagnostic tests (RDTs) for field diagnosis, especially in remote areas.
- In individuals who test positive for the treponemal antibody, use the non-treponemal line of a point-of-care test (e.g., the Chembio DPP Screen and Confirm test) and initiate treatment in the case of positivity.
- Whenever possible, use laboratory-based tests, such as rapid plasma reagin (RPR), to distinguish between past-treated and current infection.
- Interpret a high-titer non-treponemal RPR test result (≥1:8) (or the corresponding DPP result) as an indicator of current infection requiring intervention.
- Consider the age and timing of testing when interpreting test results:
  ○ In young children born after the provisional transmission interruption date, a positive treponemal test alone (in the absence of a history or stigmata of congenital syphilis) is reliable for identifying current infection and can be utilized to verify transmission in this age group.
  ○ In children born before the provisional transmission interruption date, a positive treponemal test needs confirmatory testing with non-treponemal assays.
- Ensure quality control measures are in place to maintain test accuracy and reliability.

**3. Recommendations for designing yaws-specific serosurveys**

**3.1 Survey timing**

- Conduct initial serosurveys at least 2 years after the last yaws case was reported (i.e., the provisional transmission interruption date).
- Plan at least two follow-up serosurveys, each separated by at least 1 year or more, over a period of 3–10 years to monitor for ongoing transmission, contingent upon the local capacity to conduct serosurveys.

**3.2 Selection of evaluation units (EUs), target population, and required sample size**

- Choose EUs based on local yaws epidemiology, programmatic feasibility, and an all-age population size per EU of up to 500,000.
- Estimate the total number of children aged 1–5 years, who constitute the target population, living in the EU.
- Use Table 2 to determine the required sample size based on the total target population in the EU.

**3.3 Selection of primary sampling units and households**

- Identify all primary sampling units (PSUs, villages or equivalent) within the EU.
- In EUs with ≤30 PSUs, survey all PSUs.
- In EUs with >30 PSUs, select a sample of PSUs that is representative of the population at risk, therefore minimizing bias in the estimate of EU-wide yaws prevalence:

**3.4 Georeferencing**

- Collect Global Positioning System (GPS) coordinates for each selected households in the PSU.
- Consider the use of geospatial analysis to enhance the precision of prevalence estimates and identify areas needing focused interventions, e.g., by identifying areas (e.g., sub-districts, or 5 × 5 km pixels) where we are >95% certain that the prevalence is >1%.

**4. Recommendations for designing integrated yaws serosurveys**

**4.1 Survey integration**

- Identify existing community-based survey platforms, such as trachoma surveys or lymphatic filariasis transmission assessment surveys, into which collection of data on yaws could be integrated.
- Ensure the chosen instrument has the potential to support recruitment of children aged 1–5 years.

**4.2 Sample size and representation**

- Aim for a minimum sample size of 1,000 children aged 1–5 years per EU.

**4.3 Sampling design**

- Use the sampling methods already established for the primary survey platform and adapt to add the yaws survey.

**4.4 Georeferencing**

- Same as in 3.4

**4.5 Coordination and training**

- Ensure appropriate integration of yaws surveillance and train survey teams on the additional procedures required for yaws testing and data collection.

**5. Recommendations for follow-up surveys**

- Conduct at least two follow-up surveys, each separated by at least 1 year or more, over 3–10 years to confirm ongoing <1% yaws seroprevalence and monitor for transmission re-emergence.
- Use model-based geostatistical methods to account for spatial heterogeneity in yaws prevalence.
- Tailor follow-up surveillance surveys based on local context and initial survey results.
- Possible aims for the follow-up surveys include:
  ○ Conduct follow-up surveys to measure if the mean predicted prevalence of yaws is below 1% with greater precision, particularly if the initial survey lacks adequate statistical power.
  ○ Focus follow-up sampling in areas with the greatest uncertainty about threshold achievement if the initial survey shows the <1% threshold is reached.
  ○ Intensively sample administrative areas where the estimated prevalence exceeded 1% in the initial survey and received targeted interventions to check no new seropositive cases.
- Perform additional follow-up surveys if any survey finds yaws prevalence above 1% across the EU.

**6. Recommendations for handling positive tests during serological surveys**

- Confirm positive treponemal RDT results with non-treponemal DPP testing in the field or RPR tests in the lab, if venous blood collection is feasible.
- For EUs with confirmed infections, conduct intensified serological sampling and active clinical case searches, using PCR confirmatory testing when possible.
- Assess RPR titers, demographic and clinical data to distinguish between current versus past-treated infections.

**7. Recommendations interpretation of survey results**

- Analyze yaws serosurvey prevalence to see if the upper 95% confidence limit is below the 1% threshold.
- If population seroprevalence exceeds the threshold (>1%) at any time point, two additional yearly serosurveys and active case searches should be added to the surveillance period.
  ○ If more latent cases are found, consider a new treatment intervention and follow with three more serosurveys.
  ○ If active yaws cases are confirmed, deploy a new MDA intervention and continue surveillance until zero active cases are achieved.
- Consider serosurveillance data, along with other passive and active surveillance programs, for making community treatment decisions.

**Table 1:** List of available serological tests for trepanomatoses, including yaws [16–18].

Assay	Advantages	Disadvantages	Sensitivity (Se)/Specificity (Sp)
Laboratory-based serological tests: Treponemal tests (TPPA/TPHA/EIA)^1^ Non-treponemal tests (RPR/VDRL)^2^	Established and well-characterized Widely available Defined serological titers for RPR/VDRL High throughput for TPPA/EIA	Require venipuncture Require transportation of specimens to the laboratory, including cold chain, and trained laboratory personnel, increasing cost and time-to-result and potentially decreasing the proportion of positive results tracked back to source	Reference-standard
Point-of-care tests: Sequential use of a treponemal test followed by the DPP Syphilis Screen and Confirm^3^ non-treponemal line	Treponemal tests are widely available for syphilis programmes Can be stored at room temperature Ease and rapidity of use; can be done anywhere Performed on fingerprick blood samples Minimal training required Semi-quantitative non-treponemal result via DPP reader Avoids the need to revisit communities for follow-up (important when access is difficult or expensive)	DPP tests are more expensive than laboratory-based tests, and there is only one manufacturer Cannot be multiplexed with other assays, except for HIV serology with treponemal tests. Time delay in the field (~15–30 min) DPP reader improves performance, but is expensive and therefore not always used	Performance of DPP test: – RPR ≤ 1:8 T Se 74%/Sp 97% NT Se 60%/Sp 95% – RPR ≥ 1:16 T Se 98%/ Sp 80% NT Se 97%/Sp - Note: T, treponemal line, NT, non-treponemal line
Bead-based immunoassay: Rp17 TmpA	Fingerprick blood sample or dried blood spots Semi-quantitative result High throughput Can be multiplexed with other assays on the same sample May be particularly practical if a strategy is adopted that involves centralized testing, there is integrated serosurveillance across multiple programmes or for ‘confirmatory’ testing across countries	Laboratory-based testing Available only in reference laboratories in some countries Mostly treponemal test (e.g., Rp17) Result rarely available on day of sampling Does not rule out prior, treated infection	Rp17 v TPPA Se 90%/Sp 97% TmpA v RPR Se 80%/Sp 98% Note: TmpA may be used as a potential proxy marker for recent or active infection

^1^TPPA (*Treponema pallidum* particle agglutination), TPHA (*Treponema pallidum* hemagglutination assay), and EIA (enzyme immunoassay) are “treponemal” (T) tests. They are qualitative and generally remain positive for life in an individual who has been infected.

^2^RPR (rapid plasma reagin) and VDRL (venereal disease research laboratory), are “non-treponemal” (NT) tests (also known as antilipoidal antibody tests). They can be non-specifically positive due to other inflammatory processes, and therefore are only of significance if they are accompanied by a positive “treponemal” test. Can be quantitative, by undertaking a dilution series. Titer declines with successful treatment and should eventually become negative.

^3^DPP, dual path platform, a commercial rapid test that incorporates both a treponemal and non-treponemal assay.

## Delineation of evaluation units

EUs must be selected by considering local yaws epidemiology, programmatic feasibility, and population size:

i) Epidemiology: The risk of yaws should be relatively homogenous within each EU. Factors such as historic yaws data, geography, population density, socio-economic status, and the degree of remoteness or rurality could be considered for assessing risk homogeneity. Neighboring communities and districts not known to have previously been endemic should also be considered for inclusion in serosurveys, especially if they have similar risk factors as the endemic area. In areas with a history of many cases of yaws or in sub-populations that could sustain yaws transmission independently of the rest of the population (e.g., island communities or mobile populations), relatively smaller EUs may be necessary. While more costly, defining smaller EUs with the same sample size leads to greater sampling density and reduces the chance that serosurveys fail to detect evidence of infection that is present in the population.
ii) Feasibility: The selected EU should correspond to a geographical area where public health interventions can be effectively delivered: ideally a province, district or sub-district. An advantage of using districts as the EU is that doing so facilitates alignment with population-based surveys conducted for other indications. Due to limited resources for single-disease assessments, national yaws programmes need to be flexible and should try to integrate with other serosurveys, such as trachoma surveys [20], transmission assessment surveys for lymphatic filariasis [21], vaccine-preventable disease surveys, community- or school-based nutritional surveys or population-based HIV impact assessments as much as possible. The national programmes must carefully consider these epidemiological and practical factors when defining the EUs for yaws serosurveys and deciding on appropriate integration opportunities.
iii) Population size: The population size of each EU should be carefully considered to facilitate effective survey administration and efficient resource allocation. For a 30-cluster survey, it is recommended that EUs maintain a maximum population of 500,000 and have a similar size within a given country. While a precise minimum size has not been established, total population for surveys usually range from 100,000 to –250,000 [22], with lower sizes (20,000–50,000) suggested to facilitate decision-making [7].

## Yaws-specific initial serosurveys

### Selection of primary sampling units and households within a PSU

The initial serosurvey targets children aged 1–5 years who reside in the EU. PSUs are the smallest administrative units within the EU, such as villages, communities, neighborhoods, or census enumeration areas, for which a complete list is available. Table 2 lists the required sample sizes for different scenarios: in EUs with ≤30 PSU (all PSUs are included) and in EUs with >30 PSUs (cluster sampling is recommended).

**Table 2:** Number of children to be tested for yaws-specific initial serosurvey.

Expected population size^*^ of children 1–5 years-old in the EU	All PSUs included	Sampling of PSUs
	Required sample size^*^ (*n*)^1^	Required sample size^*^ (*n*)^2^
<1,000	Census^3^	NA^4^
1,000–1,999	726	NA^4^
2,000–4,999	1,034	3,102
5,000–7,999	1,370	4,110
8,000–13,999	1,400	4,200
14,000–9 999	1,550	4,650
30,000–59,999	1,558	4,674
≥60,000	1,580	4,740

*Expected population size = number of individuals estimated to be resident within the evaluation unit (EU). Required sample size = target number of individuals to enroll in the survey.

^1^Sample size calculations were conducted using the hypergeometric distribution. The rate of type 1 error (falsely concluding that the prevalence is <1% when in truth it is ≥1%) was set at 0.05 and the rate of type 2 error (falsely concluding that the prevalence is ≥1% when in truth it is <0.5%) was set at 25%.

^2^For cluster-based sampling, a design effect of 3.0 was assumed.

^3^Include in the survey all children aged 1–5 years living in the EU in the survey.

^4^Not applicable; the total population size is too small to merit a cluster survey, take all primary sampling units instead.

**Fig 2:**
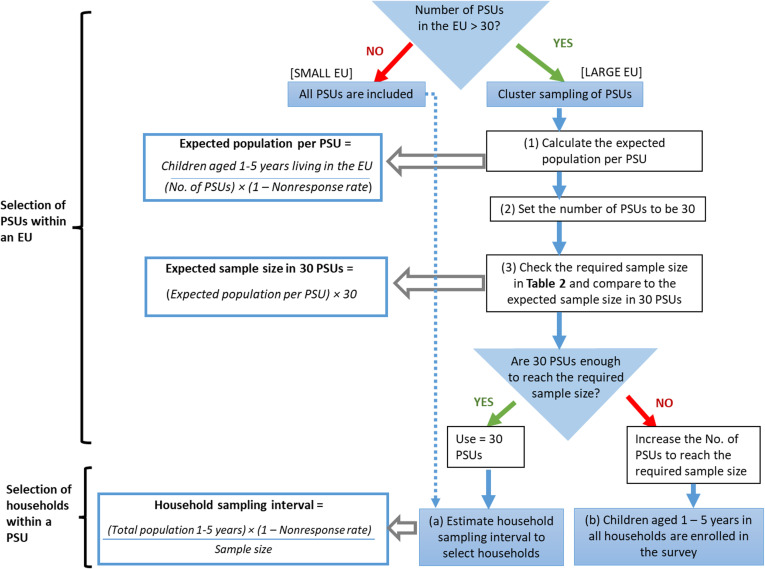
Algorithm for determining the number and selecting primary sampling units and households.

Following this, Fig 2 provides a flowchart for decision-making in the selection of PSUs and households within each PSU.

In small EUs with ≤30 PSUs, all PSUs need to be included in the survey. For the selection of households, a household sampling interval should be established. The survey team and local leaders should use this interval to plan a route that covers all households. At each selected household, the survey team will enroll all children aged 1–5 years until all households in the plan are accounted for. The S1 Appendix provides guidelines and an example for estimating the household sampling interval.

In large EUs with >30 PSUs, cluster sampling of PSUs is needed, which involves randomly selecting a subset of PSUs to survey. The steps displayed in Fig 2 for cluster sampling in large EUs are further described in the S1 Appendix.

In all cases, survey teams should be asked to record the GPS coordinates of the selected households in each of the PSUs visited. These data, combined with the serosurvey results, will guide subsequent follow-up surveys.

## Yaws initial serosurvey integrated into existing programs

Integration of serosurveys into existing programs is an opportunity to optimize public health resources, with potentially important benefits for the population. To assess whether an existing survey can be adapted to integrate yaws transmission interruption assessments, consider the survey scale (ensuring it represents the entire EU) and the demographics of the target population. School-based surveys generally miss preschool-age children, who are crucial for yaws assessments, making household-based surveys more suitable due to their broader age coverage. National programmes may wish to explore potential platforms for integration considering the key recommendations in Box 1.

For example, in trachoma impact and surveillance surveys that include all residents of selected households aged 1 year and above, yaws testing could include all selected 1–5-year-olds, with consideration given to adding households to exceed 1000 individuals aged 1–5 years [23]. In the case of lymphatic filariasis Transmission Assessment Surveys, typically targeting children aged 6–7 years, children aged 1–5 years in all selected households may be invited to undergo yaws testing. In a lymphatic filariasis IDA Impact Surveys, which target adults aged ≥20 years, yaws testing could extend to all households selected, enrolling children aged 1–5 years. Other survey platforms may include those for vaccine-preventable diseases, non-communicable diseases, or malaria. Table A in S1 Appendix provides detailed information regarding ongoing survey platforms that could integrate yaws serosurveys.

## Follow-up surveys

The initial serosurveys aim to provide a geographically representative estimate of yaws seroprevalence across the entire EU. However, the design does not take into account the spatial heterogeneity in yaws prevalence. Model-based geostatistical methods yield more precise predictions of EU-wide prevalence and quantify within-EU heterogeneity by incorporating spatial correlation and environmental covariate information from georeferenced data collected during the initial serosurvey [24] (further explanation and theoretical framework for model-based geostatistics are provided in the S1 Appendix). Whereas initial surveys may be integrated with other surveys, follow-up surveys may need to be tailored based on baseline findings and may not be as amenable to integrating.

It is suggested that the national programmes consider the serosurvey objectives, the local context, the initial survey (Survey ‘S1’ in Fig 1) results, and the recommendations in Box 1 to inform the follow-up survey design (Survey ‘S2’, ‘S3’, etc. in Fig 1). The aims of these follow-up surveys may vary according to the estimated seroprevalence in the EU at the initial serosurvey. Examples of possible aims for follow-up surveys are provided in the S1 Appendix.

At least two follow-up surveys should be conducted to demonstrate with high confidence that the threshold has been achieved and maintained over time and that there are no signs of re-emergence. While these surveys may be conducted annually, it may be preferable to space them out over several years to enhance the detection of disease re-emergence.

## Handling positive tests during serological surveys

**Fig 3:**
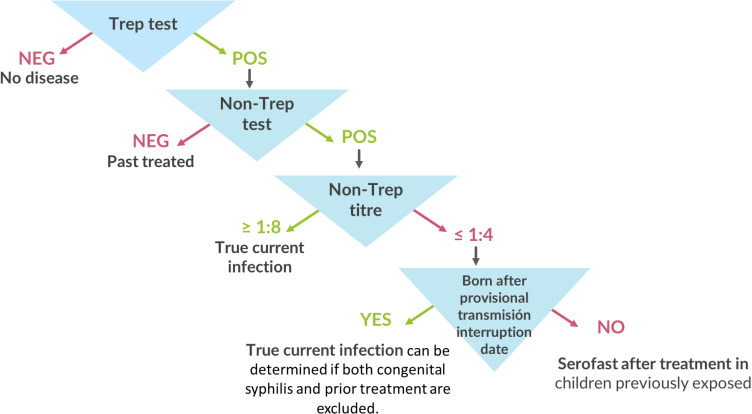
Testing and decision algorithm for determining infection in individual positive results during serological survey. Trep: Treponemal test; Non-trep: Non-treponemal test; POS: Positive NEG: Negative. ^a^Given the reactivity of serological tests to syphilis, this algorithm does not apply to children with a history of congenital syphilis.

When asymptomatic children in a village test positive for yaws during a serosurvey, it is important to understand whether they have current infection. Fig 3 summarizes the decision algorithm for classifying a case as a true current yaws infection versus past treated cases.

In the field, individuals should be tested using a treponemal RDT, which should then be verified with the non-treponemal line of a DPP assay to distinguish current infections from past treated cases using a finger prick. In the laboratory, positive treponemal tests can be confirmed with RPR tests, which require a venous sample. High RPR titers (≥1:8) suggest current infection, either active or latent; low titers (≤1:4) should be interpreted variably, based on birthdate: past treated infection (i.e., serofast) in children born before the zero-case declaration, and recent infection in those born after the zero-case declaration. Treatment with azithromycin can be initiated upon treponemal RDT positivity since recalling patients for treatment may be difficult in many environments. However, confirmatory testing is important for interpreting programmatic results and making informed decisions. Actions to follow include:

1. For confirmed current infections, intensified serological sampling (i.e., repeated testing to the same individual for investigating serological progression and sampling household contacts to investigate local transimission) and active clinical case searches should be conducted, with PCR testing used for clinical cases whenever feasible.
2. For potential serofast cases, where children have low titers, but are known to have been treated, interviews with parents or guardians can provide the child’s history and symptoms.
3. In children with low RPR titers who were not previously treated, appropriate treatment for yaws should be administered and intensified sampling and clinical searches should be reinitiated.
4. If no new cases are found despite intensified sampling, the initial findings may be false positive test results. This is especially likely as the predictive power of a positive test decreases with yaws prevalence decrease.
5. Identifying additional positive cases during intensified sampling can lead to an overestimation of the true area-wide prevalence. A statistician should analyze the data considering the sampling strategy, but if expert analysis is unavailable, survey analyses can exclude results from areas of intensified sampling for a less biased estimate.

## Recommendations for serosurvey results interpretation and decision-making

The goal of analyzing the results of yaws serosurveys is to compare the sampled prevalence with the target threshold of 1%. To determine whether the true prevalence in the population is likely to be below this threshold, it is common to report the upper bound of the one-sided 95% confidence limit for true prevalence. If this upper limit is less than 1%, it suggests that the true prevalence is probably below the target threshold. The analysis should account for the survey design; in particular, it is important to account for clustering of positives if a cluster-based survey design was used.

If any initial or follow-up surveys find yaws prevalence above 1%, two additional years of serosurveys and active case searches should be added to the surveillance period. If latent yaws cases (asymptomatic seroreactors) are still found during this period in children aged 1–5 years, a new treatment intervention (TTT or TCT) should be considered, followed by three more rounds of serosurveys. If active yaws cases (symptomatic cases) are detected and confirmed by serology and PCR during active case searches, a new intervention (TTT or TCT) should be deployed, and surveillance should continue until zero active cases are achieved, followed by seroprevalence surveys starting anew. The intervention choice should be made based on the WHO guidelines in force [4]; however, consideration of evolving evidence regarding the effectiveness of TCT followed by TTT versus repeated TCT rounds is recommended [10,25]. In case TTT or TCT is needed, close monitoring of treatment failures is essential to identify macrolide resistances [11,26].

Of note, decisions regarding the epidemiological progress of yaws in a given area, should be make by combining the information from serosurveys and passive and active surveillance, when applicable.

## Conclusion

This policy paper for yaws serosurveys provides guidance on a comprehensive approach to sequential serosurveys required to confirm the interruption of yaws transmission over time. Ensuring sufficient sample sizes and systematic PSU selection, coupled with georeferenced data, enhances the precision of prevalence estimates and facilitates targeted interventions. Positive serological results should be confirmed with additional testing, and high-titer reactions should trigger intensified surveillance and intervention, including PCR testing for symptomatic individuals.

While all recommendations in this paper are evidence-based and stem from extensive experience in yaws management, caution is advised when applying them. First, the sample size calculations and recommendations reflect the current global standard but should be adapted to local contexts by experts; larger sample sizes are encouraged when possible, given the goal of yaws’ eradication. Second, no single survey can confirm transmission interruption in a population. Health ministries should gather multiple lines of evidence, such as surveillance records and consecutive serosurveys over several years, to ensure confidence in transmission interruption. Third, some critical recommendations lack robust evidence but are provided to assist initial program development. As more data emerges from yaws serosurveys, guidance may be updated; users should stay informed of any revisions or new recommendations.

For local yaws elimination efforts to succeed, sustained political support, targeted funding, ongoing training for health workers, maintaining passive surveillance, and active testing are indispensable. Equally crucial is engaging communities with high-level awareness about yaws and implementing and maintaining robust monitoring and evaluation frameworks to assess the effectiveness of intervention and surveillance strategies. Advancing the goal of yaws eradication globally also requires continuous research and global partnership and inter-sectoral collaboration among stakeholders.

## Supporting information

S1 Appendix Supporting information file. **Table A.** Examples of routine survey platforms that may provide opportunities for yaws integration.
(PDF)
