# Supplementary material for: Guidance for conducting and evaluating serological surveys to assess interruption of yaws transmission in the context of an eradication target
Source: PLoS Negl Trop Dis. 2025 Apr 23;19(4):e0012899. doi: 10.1371/journal.pntd.0012899 (PMC12017476; doi:10.1371/journal.pntd.0012899)
Supplement: S1 Appendix — Table A. Examples of routine survey platforms that may provide opportunities for yaws integration. (PDF) [file pntd.0012899.s001.pdf]

Serological Survey to Assess Interruption of Yaws Transmission in the Context of Eradication

Supplementary Appendix

CONTENTS

Contents 1

1 Glossary of terms 2

2 Estimate of the household sampling interval 5

2.1 Instructions 5

2.2 Example 5

3 Cluster sampling (no more than 30 clusters) 6

3.1 General instructions for selecting PSUs 6

3.2 Household sampling interval 7

3.2.1 Example 7

4 Theoretical framework for the use of model-based geostatistics 9

5 Examples of possible aims for the follow-up survey 11

6 Supplementary Tables 12

7 Reference 13

1 GLOSSARY OF TERMS

Yaws Clinical Presentation:

Suspected yaws case: A person of any age who is or was living in a previously or currently endemic area and who presents with clinical signs consistent with yaws.

Serologically confirmed yaws case: A case of suspected yaws with a positive treponemal and non-treponemal test.

PCR confirmed yaws case: A case of suspected yaws that is positive in Treponema pallidum polymerase chain reaction (PCR).

Classification based on laboratory and field assays:

Person with past yaws infection: Someone with serology results showing a reactive treponemal test and a non-reactive non-treponemal test; may be asymptomatic or with symptoms caused by other skin ulcer pathogens.

Person with current yaws infection: Someone with positive results from both treponemal and non-treponemal serological tests; may be asymptomatic (latent yaws), symptomatic with symptoms caused by the treponemal infection (active yaws), or symptomatic with symptoms caused by other skin ulcer pathogens.

Person with latent yaws infection: Someone who tests positive on both treponemal and non-treponemal serological tests but does not currently exhibit yaws-like clinical symptoms, or alternatively, presents yaws-like clinical symptoms but receives a negative T. pallidum PCR result on a lesion swab.

Person with active yaws infection: Someone who tests positive on both treponemal and non-treponemal serological tests and presents with yaws-like clinical symptoms along with a positive T. pallidum PCR on a lesion swab.

High-titre non-treponemal reaction: Typically defined as an RPR (Rapid Plasma Reagin) titre ≥ 1:8, or an equivalent DPP NT (Dual Path Platform Non-Treponemal) value, which is usually associated with a true current (active or latent) infection.

Low-titre non-treponemal reaction: Typically defined as an RPR(Rapid Plasma Reagin) titre ≤ 1:4, or an equivalent DPP NT value. In children previously exposed to a treponemal infection, a low-titre reaction may sometimes be associated with serofasting, wherein positive results on serological tests persist despite receiving appropriate treatment and being cured.

Epidemiological Terms:

Active case detection: proactive approach aimed at actively identifying individuals who may present active yaws conducting systematic screenings, outreach efforts, or targeted surveillance activities within a population or community. Implemented by health workers or village health workers.

Elimination (or interruption of transmission): reduction to zero of the incidence of infection caused by a specific pathogen in a defined geographical area, with minimal risk of reintroduction, that is achieved as a result of deliberate efforts. Continued action to prevent re-establishment of transmission may be required.

Eradication: permanent reduction to zero of the worldwide incidence of infection caused by a specific pathogen as a result of deliberate efforts, with no risk of reintroduction. This definition is used at global level

Evaluation Unit (EU): the geographical unit where yaws endemicity or prevalence will be assessed to trigger a response.

Model-based geostatistics (MBG): a statistical approach that combines georeferenced prevalence survey data with raster data on natural and/or social environmental risk-factors to make predictions about prevalence at sampled or unsampled locations, and at EU-level.

'Post-zero case phase': refers to the period following the reduction of yaws to zero reported cases. During this phase, efforts focus on surveillance to ascertain interruption of transmission.

Primary sampling unit (PSU): the first administrative unit that is sampled in a survey (typically corresponds to a village, community or census enumeration area)

Seroprevalence: the percentage of population presenting antibodies against a given pathogen.

Serosurvey: the collection and testing of blood samples from a population to measure the prevalence of antibodies against a given pathogen.

Technical Advisory Group: a committee whose aim is to support the national yaws interruption of transmission effort, comprised of individuals with the epidemiological, clinical and statistical expertise required to advise the MOH on the timing, design and interpretation of yaws serosurveys

2 ESTIMATE OF THE HOUSEHOLD SAMPLING INTERVAL

2.1 INSTRUCTIONS

1. Estimate the household sampling interval by applying the following formula:

$$\text{Houshold Sampling Interval} = \frac{(\text{Total population 1-5 years}) \times (1 - \text{Nonresponse rate})}{\text{Sample size}}$$

- Total population 1-5 years: Estimated number of children in the target age group living in the evaluation unit (should be based on the most recent population projections)
- Nonresponse rate: Proportion of 1-5-year-olds who will not provide a valid specimen due to either absence, parental refusal, serious illness, or any other factor. If unknown, use at leats 15% (i.e., 0.15)
- Sample size: As estimated from **Table 2** based on the decision algorithm in **Figure 2**

2. Select a random number between 0 and the HH Sampling Interval and then round up to the nearest whole number to determine the starting household.
3. Sequentially add the HH sampling interval to select additional households, rounding up each time.
4. Upon arriving at each sampling unit, the survey team and local leader should plan a route to cover all households.
5. The survey team an local leader should follow this route and stop at each household and enrol all children aged 1-to-5 years. This household should be marked and counted. The recruitment should proceed until all households in the plan are counted.

2.2 EXAMPLE

For example, if the household sampling interval is 4.32, a random number generator might return 0.87. Rounding up to the nearest whole number results in '1' being the starting household.

The subsequent adding of household numbers would result in the following table

HH Sampling Interval	Selected household
0.87	1
5.19	6
9.51	10
13.83	14
18.15	19

Random start → 0.87

Random start + HH sampling interval (0.87 + 4.32) → 5.19

3 CLUSTER SAMPLING (NO MORE THAN 30 CLUSTERS)

3.1 GENERAL INSTRUCTIONS FOR SELECTING PSUS

In large EUs, the first step is to determine the number of PSUs needed for the survey:

1) Calculate the expected number of children aged 1—5 years old per PSU that should be invited to participate in the survey (the “expected target population size per PSU”). To do this, first determine the anticipated non-response rate, which is related to absence, parental refusal, serious illness, or any other factor. If unknown, use at least 15% (0.15). The expected target population size per PSU is then determined by: $\frac{Total\ number\ of\ children\ 1{-}5\ years\ living\ in\ EU}{(Number\ of\ PSU\ in\ EU) x\ (1 - non{-}response\ rate)}$
2) Set the minimum number of PSUs needed as 30.
3) Determine if 30 PSUs will be sufficient to reach the sample size (Table 2, column 3) by comparing it to the expected sample size from the 30 clusters ([expected target population size per PSU] × 30). If the expected sample size that would be achieved from visiting 30 PSUs is less than the sample size required in the Table below, then additional PSUs are needed. In such instances, the number of PSUs should be increased to [Sample size / Expected target population size per PSU] and rounded up to the nearest whole number.

Number of children to be tested for yaws-specific initial serosurvey (reproduced from Table 2 in the body text)

**Expected Population Size* of children 1-5 years-old in the EU**	**All PSUs included**	**Cluster-based sampling of PSUs**
	**Required sample size* (n)[3]**	**Required sample size* (n)[4]**
<1000	Census[1]	NA[2]
1000-1999	726	NA[2]
2000 - 4999	1034	3102
5000 - 7999	1370	4110
8000 – 13 999	1400	4200
14 000 – 29 999	1550	4650
30 000 – 59 999	1558	4674
>60 000	1580	4740

* Expected population = number of individuals anticipated during the survey. Required sample size = specific number aimed for inclusion.

[1]Include all children aged 1-5 years living in the EU in the survey.

[2]Not applicable; the total population size is too small to merit a cluster survey, take all PSUs instead.

[3]Sample size calculations were conducted using the hypergeometric distribution. The rate of type 1 error (falsely concluding that the prevalence is <1% when in truth it is >1%) was set at

0.05 and the rate of type 2 error (falsely concluding that the prevalence is $\geq$1% when in truth it is <0.5%) was set at 25%.
[4]For cluster-based sampling, a design effect of 3.0 was assumed.

For household selection in large EUs, depending on the final number of PSUs needed to reach the target sample size, two scenarios may arise:

a) When the number of children in 30 PSUs is sufficient to achieve the required sample size and exceeds what is actually needed, only a proportion of the households within each PSU needs to be included. A household sampling interval can be used to select which households to include.
b) When more than 30 PSUs are needed to achieve the required sample size, which would result when the average number of children in each PSU is small or the nonresponse rate is high, then, all households in the PSUs are included in the survey (and all children in the target age group living within these households are invited to participate).

3.2 HOUSEHOLD SAMPLING INTERVAL

1. Estimate the within-cluster sampling interval using the following formula:

$$Within\ Cluster\ Sampling\ interval = \frac{Expected\ target\ pop.per\ PSU * 30}{sample\ size}$$

2. Select a random number between 0 and the HH Sampling Interval and then round up to the nearest whole number to determine the starting household
3. Sequentially add the within-cluster sampling interval to select additional households, rounding up each time.
4. Upon arriving at each sampling unit, the survey team and local leader should plan a route to cover all households.
5. The survey team an local leader should follow this route and stop at each household and enrol all children aged 1-to-5 years. This household should be marked and counted. The recruitment should proceed until all households in the plan are counted.

3.2.1 Example

For example, if the sample size is 4200 and the expected target pop. per PSU is 160, then the within cluster sampling interval is 1.14. If a random number between 0 and 1.14, like 0.22 is chosen, rounding up means starting with household #1.

If we add the sampling interval (1.14) to the random number (0.22) the result if 1.36. Rounding up gives us 2. The second household to be selected would be household #2.

Random start

Random start + HH sampling interval (0.22 + 1.14)

Within Cluster Sampling Interval	Selected household
0.22	1
1.36	2
2.5	3
3.64	4
4.78	5

4 Theoretical framework for the use of model-based geostatistics

Model-based geostatistics can provide more accurate prevalence estimates compared to traditional sampling methods [1-3]. This approach leverages spatial correlation, where prevalence measured at one sampled location helps predict prevalence at nearby unsampled locations within a certain distance range. In the case of yaws prevalence, spatial correlation is anticipated due to the infectious nature of the disease, which leads to clustering of cases in geographic proximity.

The following Figure corresponds to the prevalence survey conducted in the Solomon Islands [4]. In this case, spatial correlation operates over a range of coastal distances estimated to be in the tens of kilometres (point estimate for the Solomon Islands dataset 25.7 km, approximate 95% confidence interval 6.5–100.8). In practice, spatial correlation depends on local factors (e.g., ease of access, transportation routes, human behaviour) in addition to biological parameters (e.g., transmissibility). For this reason, it is unwise to apply model-based geostatistics automatically; expert guidance should be sought.

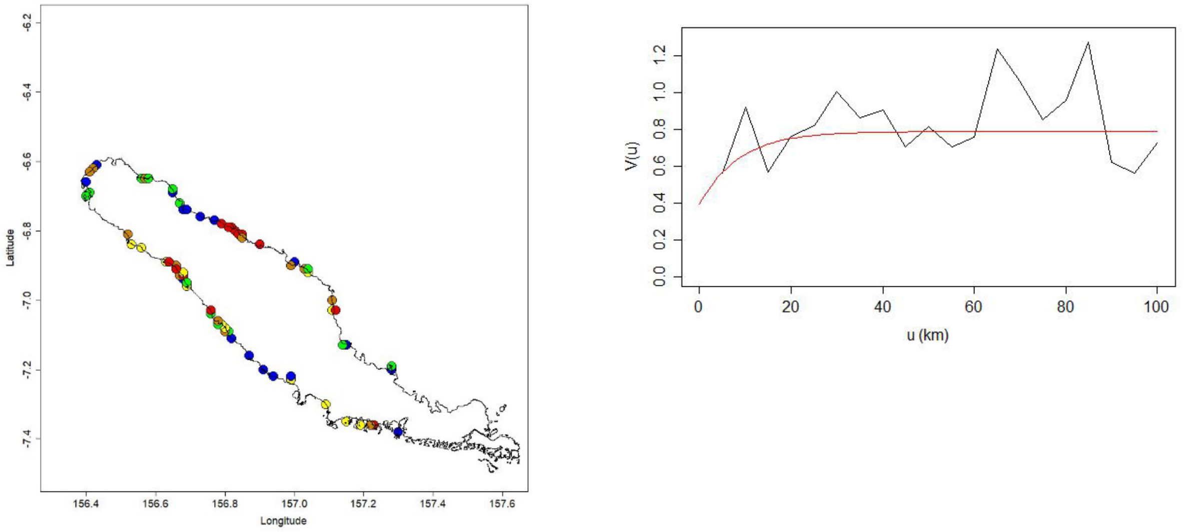

Figure. The left-hand panel shows sampling locations on Choiseul, Solomon Islands, colour-coded by quintiles of measured yaws prevalence, from blue (lowest) through green, yellow and brown to red (highest). The right-hand panel shows the variogram, a graphical tool to estimate spatial correlation; an increasing variogram indicates correlation decreasing as the coastal distance between locations increases. The black line is the empirical variogram of the data in the left-hand panel, the red line is the variogram of a fitted model in which correlation, r, is assumed to decrease as coastal distance, u, increases, according to the equation $r(u) = \exp(-3u/\phi)$. The parameter ϕ is the estimated range of the spatial correlation.

Currently, the spatial correlation of yaws is not well understood for most endemic populations, making it challenging to apply model-based geostatistics when designing the first serosurvey in an evaluation unit. However, data from this initial serosurvey could inform subsequent surveys by accounting for spatial variability and focusing on areas of high uncertainty or previously high

prevalence. Although these follow-up serosurveys might be harder to integrate, the possibility of combining second, third, and future yaws serosurveys with other surveys should not be ruled out. Often, discussions about design principles and practical requirements reveal shared opportunities for integration

5 EXAMPLES OF POSSIBLE AIMS FOR THE FOLLOW-UP SURVEY

The following examples illustrate potential scenarios in which a follow-up survey might be necessary:

1. If the initial survey lacks sufficient power to demonstrate that the seroprevalence is below 1% using the upper bound of a one-sided 95% confidence interval (CI), the follow-up survey may aim to measure whether the mean predicted seroprevalence in the EU is below 1% using a model-based geostatistical design, which is likely to offer greater precision.
2. If the initial survey indicates that the <1% threshold has been reached at the EU level, the follow-up survey could employ a model-based geostatistical design to resolve uncertainty about threshold achievement; for example, in an area where the predictive probability of prevalence is close to 0.5 (50%), taking additional samples in this area would drive the predictive probability closer to either zero or one, according to whether, respectively, the additional samples did or did not detect additional cases.
3. If the initial survey identifies an administrative area with an elevated number of cases (prevalence > 1%) that subsequently received targeted action, such as focal treatment, follow-up surveys may intensively sample these areas to determine if the local prevalence has decreased. In this scenario, TTT or TCT interventions should be considered.

6 SUPPLEMENTARY TABLES

Table S1. Examples (¥) of routine survey platforms that may provide opportunities for yaws integration

	**Trachoma Impact & Surveillance Surveys (TIS & TSS)**	**Lymphatic Filariasis Transmission Assessment Surveys (TAS)**	**Lymphatic Filariasis IDA Impact Survey (IIS)**
**Evaluation unit**	District	District	District
**Primary sampling unit**	Community	School or Community	Community
**Number of PSUs**	20 - 30	≥30	30
**Sampling of PSUs**	Random (using probability proportionate to estimated size sampling)	Random (systematic sampling from geographically ordered list)	Random (using probability proportionate to estimated size sampling)
**Target Population**	Children 1-9 years	Children 6-7 years	Adults (≥20 years)
**Sample size**	~1164	~800 - 1692	~3100
**Considerations for yaws integration**			
**Feasibility**	Very feasible	Feasible if the PSU is the community; not feasible for school-based surveys	Feasible
**Rationale**	Overlapping target population makes it straightforward to add yaws testing in the 1-5-year-old participants	For household surveys, the teams would need to enrol additional children for yaws testing	The teams would need to enrol children for yaws testing
**Sampling suggestion**	Conduct yaws testing in all 1-5-year-olds selected for TIS/TSS survey; consider adding additional households per PSU to increase sample size to ≥1000 individuals ages 1-5 from EU	Conduct yaws testing in all households selected for TAS regardless of whether they have a child in the TAS age group. For each selected household enrol all children ages 1-5 in the yaws survey. This should result in a sample size of 2000 - 4000 children	Conduct yaws testing in all households selected for IIS. For each selected household enrol all children ages 1-5 in the yaws survey; consider additional households per PSU to increase the sample size to ≥1000 individuals ages 1-5 from EU

¥ The Table summarizes ongoing platforms with high penetration and sustainability; however, other platforms, such as MIS, HIAs, Epi among others may be considered.

7 REFERENCE

1. Johnson, Olatunji, et al. "Model-based geostatistical methods enable efficient design and analysis of prevalence surveys for soil-transmitted helminth infection and other neglected tropical diseases." Clinical Infectious Diseases 72.Supplement_3 (2021): S172-S179.

2. Amoah, Benjamin, et al. "Model-based geostatistics enables more precise estimates of neglected tropical-disease prevalence in elimination settings: mapping trachoma prevalence in Ethiopia." International journal of epidemiology 51.2 (2022): 468-478.

3. Sasanami, Misaki, et al. "Using model-based geostatistics for assessing the elimination of trachoma." PLOS Neglected Tropical Diseases 17.7 (2023): e0011476.

4. Marks, Michael, et al. "Mapping the epidemiology of yaws in the Solomon Islands: a cluster randomized survey." The American journal of tropical medicine and hygiene 92.1 (2015): 129.
